# Chronic Gouty Dactylitis

**DOI:** 10.31662/jmaj.2025-0357

**Published:** 2025-11-21

**Authors:** Hiroshi Shiba, Takahiro Asano, Masato Okada

**Affiliations:** 1 Department of Rheumatology, Suwa Central Hospital, Chino, Japan; 2 Immuno-Rheumatology Center, St Luke’s International Hospital, Chuo-ku, Japan

**Keywords:** gout, dactylitis, monosodium urate crystal

A 57-year-old man with a ten-year history of untreated gout presented with acute right ankle pain. Physical examination revealed multiple tophi involving the joints and subcutaneous tissues of his limbs, non-tender diffuse swelling of the fourth digit of his left hand, and arthritis of the right ankle (Figure 1A). Laboratory findings confirmed hyperuricemia and elevated inflammatory markers, including a C-reactive protein level of 4.2 mg/dL (reference, 0.0-0.3). X-rays of his hands showed erosions and a soft-tissue mass in the left fourth finger (Figure 1B). A diagnosis of an acute gout flare was established. The patient was treated with nonsteroidal anti-inflammatory drugs.

**Figure 1.**
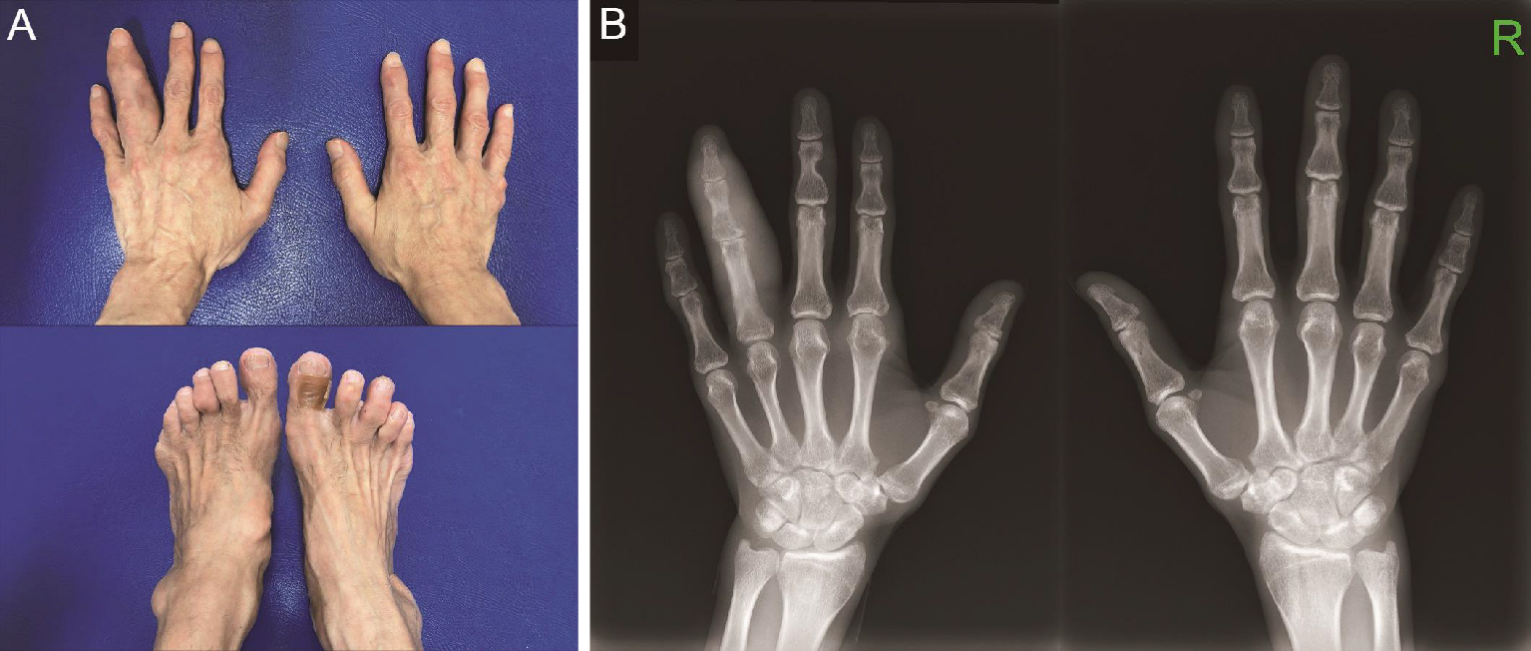
(A) Multiple tophi over bilateral hands and chronic dactylitis of the left fourth digit, and multiple tophi over bilateral feet. (B) X-rays of both hands, showing periarticular and extraarticular bone lytic lesions, and soft-tissue thickening with increased density in the left fourth digit.

In long-standing uncontrolled gout, monosodium urate crystals can deposit around tendons and phalanges, manifesting as chronic dactylitis ^(1), (2)^. The reported prevalence of dactylitis in gout is low, ranging from 5% to 10% ^(1)^. The presence of chronic dactylitis may suggest severe disease, with more tophi and involved joints, higher serum uric acid concentrations, and longer disease duration ^(2)^.

## Article Information

### Author Contributions

Data acquisition and manuscript drafting: Hiroshi Shiba. Manuscript review and supervision: Takahiro Asano, and Masato Okada.

### Conflicts of Interest

None

### Informed Consent

We have obtained informed consent for this manuscript.

### Approval by Institutional Review Board (IRB)

In this study, IRB approval was not required.
